# Chronic kidney disease and increased LAVI as risk factors of new‐onset heart failure in atrial fibrillation: A case‐control study

**DOI:** 10.1002/joa3.70061

**Published:** 2025-05-20

**Authors:** Resultanti Irwan Muin, Muhammad Yamin, Ika Prasetya Wijaya, Kuntjoro Harimurti, Hamzah Shatri, Cosphiadi Irawan, Pradana Soewondo

**Affiliations:** ^1^ Division of Cardiology, Department of Internal Medicine, Faculty of Medicine Cipto Mangunkusumo Hospital, Universitas Indonesia Jakarta Indonesia; ^2^ Division of Geriatric Medicine, Department of Internal Medicine, Faculty of Medicine Cipto Mangunkusumo Hospital, Universitas Indonesia Jakarta Indonesia; ^3^ Division of Psychosomatic, Department of Internal Medicine, Faculty of Medicine Cipto Mangunkusumo Hospital, Universitas Indonesia Jakarta Indonesia; ^4^ Division of Hematology and Medical Oncology, Department of Internal Medicine, Faculty of Medicine Cipto Mangunkusumo Hospital, Universitas Indonesia Jakarta Indonesia; ^5^ Division of Endocrinology and Metabolism, Department of Internal Medicine, Faculty of Medicine Cipto Mangunkusumo Hospital, Universitas Indonesia Jakarta Indonesia

**Keywords:** atrial fibrillation, new‐onset heart failure, risk factors

## Abstract

**Background:**

Atrial fibrillation (AF) increases heart failure (HF) risk and can eventually increase morbidity and mortality. Therefore, recognizing risk factors in AF patients is crucial to prevent heart failure. To date, there has been no research on this topic in Indonesia.

**Objective:**

To determine risk factors of new‐onset HF in AF patients.

**Methods:**

Case–control study was undertaken at Cipto Mangunkusumo Hospital using medical record data from January 2018 to May 2023. AF patients aged ≥18 years with new‐onset HF were included in the case group, and AF patients of similar age without HF were included in the control group. Patients with moderate or severe valvular heart disease, congenital heart disease, pacemakers, or implantable cardioverter defibrillators (ICD), or incomplete data were excluded. Logistic regression was used to identify significant risk factors for new‐onset HF in AF patients.

**Results:**

A total of 132 subjects consisting of 44 cases and 88 controls were included. Bivariate analysis revealed that the significant risk factors for new‐onset HF in AF patients were CAD [*p* = .037; OR 2.34 (95% CI 1.11–4.93)], CKD [*p* = .000; OR 7.78 (95% CI 3.45–17.53)], and LAVI [*p* = .002; OR 3.23 (95% CI 1.52–6.85)]. In multivariate analysis, CKD [*p* = .000; OR 6.31 (95% CI 2.69–14.77)] and LAVI [*p* = .000; OR 3.49 (95% CI 1.42–9.97)] retained their statistical significance as risk factors of new‐onset HF in AF patients.

**Conclusions:**

CKD and increased LAVI may increase the likelihood of new‐onset HF in AF patients, while hypertension, diabetes, CAD, smoking, and obesity were not significant risk factors for new‐onset HF in our study.

## Graphical abstract

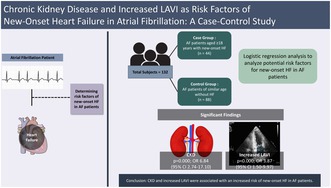

## 1 INTRODUCTION

Atrial fibrillation (AF) affects up to 33 million people worldwide, while heart failure (HF) affects about 26 million people. AF and HF have shared risk factors and can mutually lead to disease progression. These two conditions frequently coexist; the combination has a greater risk of hospitalization and mortality than either does alone.^1^, ^2^ AF causes HF through several mechanisms, including loss of atrial contractions, ventricular irregularities, tachycardia, renin angiotensin aldosterone system (RAAS) activation, adrenergic overdrive, and changes in myocardial structure and fibrosis.^1^

According to worldwide AF registries, the incidence of HF is 33% in patients with paroxysmal AF, 44% in patients with persistent AF, and 56% in patients with permanent AF.^3^ HF incidence after new‐onset AF (17.4 cases/1000 person‐years) is greater than stroke (9 cases/1000 person‐years) and myocardial infarction (4.4 cases/1000 person‐years) incidence.^4^ Consequently, specific strategies and early diagnosis are required to prevent HF in AF patients.^5^ However, studies concerning strategies for preventing HF occurrence are still limited.^6^ As such, we aimed to determine the risk factors of new‐onset HF in AF patients. We hypothesized that hypertension, diabetes, chronic kidney disease (CKD), coronary artery disease (CAD), smoking, obesity, and left atrial volume index (LAVI) were risk factors of new‐onset HF in AF patients.

## 2 METHODS

This case–control study used secondary data of AF patients collected from medical records at Cipto Mangunkusumo Hospital from January 2018 to May 2023. AF inpatients and outpatients were included. The inclusion criteria for the case group were AF patients aged ≥18 years with new‐onset HF after AF diagnoses. The inclusion criteria for the control group were AF patients aged ≥18 years without HF. The exclusion criteria were moderate or severe valvular heart disease, congenital heart disease, use of a pacemaker or implantable cardioverter‐defibrillator (ICD), or incomplete medical record data.

Of 9110 medical records of AF patients, there were 2645 AF patients without HF and 6465 AF patients with HF. We randomly selected a total of 132 subjects consisting of 44 cases and 88 controls. Controls were randomly selected using the Integer Generator (www.random.org). Cases were selected using the purposive sampling method by selecting the first 44 cases because of the limited amount of AF with new‐onset HF cases in Cipto Mangunkusumo Hospital as the tertiary referral hospital, in which most of the AF patients that come to our center usually already present with preexisting HF as a comorbidity. Logistic regression analysis with SPSS IBM 25 software was used to analyze potential risk factors for new‐onset HF in AF patients including hypertension, diabetes, CKD, CAD, smoking, obesity, and increased LAVI. Delta OR was defined as the percentage change in OR. Hypertension was defined as systolic blood pressure value ≥140 mmHg and/or diastolic blood pressure value ≥90 mmHg within two measurements, previously diagnosed hypertension, or currently taking antihypertensive medication.^7^ Diabetes was defined as a clinical syndrome along with Random Blood Glucose (RBG) value ≥200 mg/dL or Fasting Blood Glucose (FBG) value ≥126 mg/dL; RBS or 2‐hour Oral Glucose Tolerance Test (OGTT) value ≥200 mg/dL; HbA1c value ≥6.5; previously diagnosed diabetes mellitus; or currently taking insulin therapy or antidiabetic medication.^8^ Chronic kidney disease was defined as eGFR <60 mL/min/1.73 m^2^ at ≥2 visits.^9^ Coronary artery disease was defined as the evidence of coronary stenosis based on coronary angiography or imaging results.^10^ The occurrence of each potential risk factor (hypertension, diabetes, CKD, and CAD) was collected from the age of 18 years until the date of a HF incident for cases or the date of the most recent hospital visit for controls. We specified a minimum duration of all comorbidities to be 6 months. Smoking was defined as having ever smoked or still smoking at the time of data collection, and a total of at least 100 cigarettes smoked.^11^ Obesity was defined as a body mass index (BMI) of 25 kg/m^2^ or greater based on the Asian population standard from WHO.^12^, ^13^ LAVI was defined as the value at the first echocardiography examination after AF diagnosis. Increased LAVI was defined as an LAVI of more than 34 mL/m^2^.^14^ This research was approved by the Health Research Ethics Committee, Faculty of Medicine, Universitas Indonesia.

## 3 RESULTS

**FIGURE 1:**
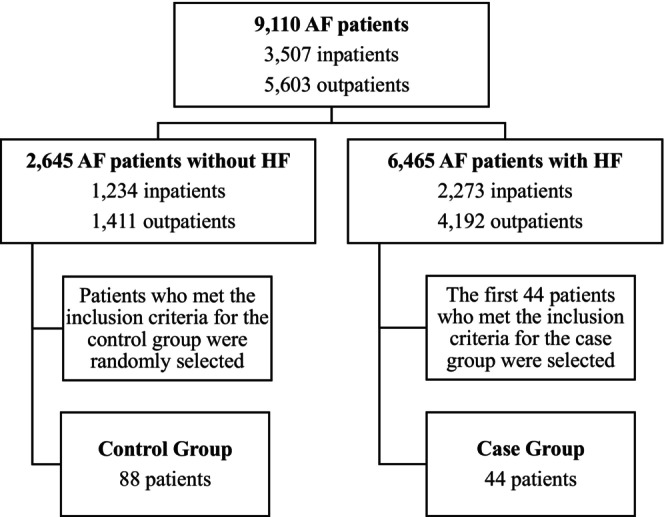
Flowchart of subject recruitment.

From a total of 9110 AF patients, 88 patients were included in the control group and 44 patients were included in the case group (Figure 1). Baseline characteristics of the subjects are presented in Table 1. Both the case and control groups were predominantly male (56.8% vs. 54.6%, respectively). This result was in accordance with other studies, which stated that AF patients comprise a greater proportion of males than females. More than half of both groups were aged ≥60 years. The most common type of AF was long‐standing persistent AF (43.2%) in the case group and paroxysmal AF (50%) in the control group. The median duration of AF in the case group was 10 months, whereas in the control group, the median was more than twofold of it (22 months). The case and control groups had median heart rates during AF of 81 and 85.5 beats per minute, respectively. More than half of the case subjects had comorbidities such as hypertension (77.3%), CAD (52.3%), and CKD (68.2%). The majority of case subjects were not diabetics (72.7%); the majority of the case and control subjects were nonsmoking (63.6% vs. 79.6%, respectively). Body mass indexes (BMIs) were similar between groups, with a median BMI of 28.5 kg/m^2^. The majority of case subjects had increased LAVI (77.3%), while 56.8% of the control group had normal values. The median values for EF baseline were similar for case and control groups (61.3% vs 61.0%, respectively).

**TABLE 1:** Baseline characteristics of case and control subjects.

Characteristics	Cases (*n* = 44)	Controls (*n* = 88)
Gender, *n* (%)		
Male	25 (56.8)	48 (54.6)
Female	19 (43.2)	40 (45.5)
Age, *n* (%)		
<60 years	16 (36.4)	25 (28.4)
≥60 years	28 (63.6)	63 (71.6)
Age, years (median, IQR)	64 (52.5–75.8)	66 (58.3–72)
Heart rate during AF, bpm (median, IQR)	81 (68.3–98.8)	85.5 (71.5–104.5)
Type of AF, *n* (%)		
Paroxysmal	18 (40.9)	44 (50)
Persistent	4 (9.1)	16 (18.2)
Long‐standing persistent	19 (43.2)	23 (26.1)
Permanent	3 (6.8)	5 (5.7)
Duration of AF, months (median, IQR)	10 (2.3–24)	22 (7.3–45)
Rhythm control on AF patients, *n* (%)		
Yes	4 (9.1)	11 (12.5)
No	40 (90.9)	77 (87.5)
Hypertension, *n* (%)		
Yes	34 (77.3)	58 (65.9)
No	10 (22.7)	30 (34.1)
Diabetes, *n* (%)		
Yes	12 (27.3)	16 (18.2)
No	32 (72.7)	72 (81.8)
CAD, *n* (%)		
Yes	23 (52.3)	28 (31.8)
No	21 (47.7)	60 (68.2)
CKD, eGFR <60 mL/min/1.73 m^2^ at ≥2 visits, *n* (%)		
Yes	30 (68.2)	19 (21.6)
No	14 (31.8)	69 (78.4)
CKD, mL/min/1.73 m^2^ (median, IQR)	50.4 (33.5–55.3)	49.5 (37.6–55.3)
Smoking, *n* (%)		
Yes	16 (36.4)	18 (20.5)
No	28 (63.6)	70 (79.6)
Duration of smoking, years (median, IQR)	30 (20–40)	21.5 (18.8–30)
BMI, *n* (%)		
<25 kg/m^2^	20 (45.5)	52 (59.1)
≥25 kg/m^2^	24 (54.6)	36 (40.9)
BMI, kg/m^2^ (median, IQR)	28.5 (26–31.5)	28.5 (26.8–30.4)
LAVI, *n* (%)		
>34 mL/m^2^	34 (77.3)	38 (43.2)
≤34 mL/m^2^	10 (22.7)	50 (56.8)
LAVI, mL/m^2^ (median, IQR)	47.7 (40.8–65.2)	48.2 (41.5–60.6)
LVEF baseline (median, IQR)	61.3 (50.0–77.1)	61.0 (50.1–85.0)

Abbreviations: BMI, body mass index; bpm, beat per minute; CAD, coronary artery disease; CKD, chronic kidney disease; IQR, interquartile range; LAVI, left atrium volume index; LVEF, left ventricular ejection fraction.

Bivariate analysis revealed that CAD, CKD, and increased LAVI were statistically significant risk factors of new‐onset HF [OR 2.34; (95% CI 1.11–4.93); OR 7.78 (95% CI 3.45–17.53)], and OR 4.47 (95% CI 1.96–10.17), respectively (*p* < .05) (Table 2). The result of multivariate logistic regression analysis revealed that CKD and increased LAVI retained their statistical significance as risk factors of new‐onset HF with OR 6.31 (95% CI 2.69–14.77) and OR 3.49 (95% CI 1.42–9.97), respectively (Table 3).

**TABLE 2:** Bivariate analysis of risk factors for new‐onset HF in AF patients.

Variable	Cases *n* (%)	Controls *n* (%)	*p*	OR (95% CI)
Hypertension			.183	1.76 (0.77–4.04)
Yes	34 (77.3)	58 (65.9)		
No	10 (22.7)	30 (34.1)		
Diabetes			.228	1.68 (0.71–3.97)
Yes	12 (27.3)	16 (18.2)		
No	32 (72.7)	72 (81.8)		
Coronary artery disease Yes	23 (52.3)	28 (31.8)	.023*	2.34 (1.11–4.93)
No	21 (47.7)	60 (68.2)		
Chronic kidney disease			.000*	7.78 (3.45–17.53)
Yes	30 (68.2)	19 (21.6)		
No	14 (31.8)	69 (78.4)		
Smoking			.049	2.22 (0.99–4.96)
Yes	16 (36.4)	18 (20.5)		
No	28 (63.6)	70 (79.6)		
Obesity			.138	1.73 (0.78–3.84)
Yes	24 (54.6)	36 (40.9)		
No	20 (45.5)	52 (59.1)		
LAVI			.000*	4.47 (1.96–10.17)
Increased	34 (77.3)	38 (43.2)		
Normal	10 (22.7)	50 (56.8)		
LVEF_baseline^a^	61.049	62.295	.351	

Abbreviation: EF, ejection fraction.

^a^ *T*‐Test.

* Significant (*p* < .05).

**TABLE 3:** Multivariate analysis of risk factors for new‐onset HF in AF patients.

Variable	*p*	OR (95% CI)
Chronic kidney disease	.000*	6.31 (2.69–14.77)
Coronary artery disease	.246	1.66 (0.70–3.93)
LAVI	.005*	3.49 (1.42–9.97)

* Significant (*p* < .05).

Interestingly, we found that the hypertension OR changed from 1.76 (95% CI 0.77–4.04) to 0.66 (95% CI 0.22–1.94) between the two analyses. Therefore, we did an additional multivariate analysis to determine the confounding factors of hypertension. We discovered that CKD was a confounding factor, with a percentage change in OR of 44.89% (Table 4). This finding may have occurred because 26 of 34 (76.5%) hypertensive patients in the case group also had comorbid CKD. This condition potentially caused the hypertension variable to lose its statistical significance after multivariate analysis.

**TABLE 4:** Multivariate analysis of hypertension with CKD and other risk factors.

Variable	*p*	OR (95% CI)	Delta OR (%)
*Crude OR*			
Hypertension	.181	1.76 (0.77–4.04)	
*Adjusted OR*			
+ Chronic kidney disease	.943	0.96 (0.37–2.50)	44.89
+ LAVI	.812	0.88 (0.32–2.39)	8.25
+ Coronary artery disease	.650	0.78 (0.28–2.20)	8.99
+ Smoking	.589	0.75 (0.26–2.12)	8.64
+ Obesity	.553	0.72 (0.25–2.08)	6.76
+ Diabetes	.451	0.66 (0.22–1.94)	4.35

## 4 DISCUSSION

Consistent with previous studies,^15^, ^16^ we had more male than female AF patients at a ratio of 1.2:1. The majority of subjects were aged more than 60 years. This finding was in agreement with reported AF population prevalences increasing with age, i.e., 0.12%–0.16% in people aged <49 years, 3.7–4.2% in people aged 60–70 years, and reaching 10%–17% in those aged >80 years. The most common type of AF was long‐standing persistent in the case group and paroxysmal in the control group, in contrast with an observational study reporting that permanent AF was the most commonly diagnosed.^16^

AF patients with CKD had a 6.84‐fold higher risk of new‐onset HF compared to those without CKD [OR 6.31 (95% CI 2.69–14.77); *p* = .000], similar to results from previous studies.^1^, ^4^, ^17^ This higher risk may have been owing to overactivation of the renin‐angiotensin‐aldosterone system (RAAS), vascular changes that can cause arterial stiffness and hypertension, and systemic inflammation in CKD patients. These conditions, along with decreased cardiac output because of the loss of atrial contraction and heartbeat irregularity in AF, can lead to HF.^1^, ^18^

We also found that AF patients with increased LAVI had a greater risk of new‐onset HF compared to those with normal LAVI [OR 3.49 (95% CI 1.42–9.97); *p* = .005]. This result was consistent with previous studies, which stated that increased LAVI was a risk factor for new‐onset HF in AF patients. Left atrium dilatation reflects the enhancement of left atrium pressure and left ventricular filling pressure. When the left atrium malfunctions and loses its buffering effects, it can lead to HF and pulmonary congestion.^19^, ^20^, ^21^

Hypertension can provoke hypertensive heart disease (HHD) marked by a prolonged increase in left ventricular filling pressure, diastolic dysfunction of the left ventricle, and left ventricular hypertrophy. These conditions are the main cause of HF in hypertension patients.^17^, ^22^ Hypertension was the most common comorbidity in our AF patients, but was not a significant factor in the incidence of new‐onset HF in AF patients based on the bivariate analysis [OR 1.76 (95% CI 0.77–4.04); *p* = .183]. This was probably because of two mechanisms: low diastolic blood pressure and decapitated hypertension. Low diastolic blood pressure disrupts coronary blood flow, leading to decreased coronary perfusion, thereby affecting the myocardium and causing HF. Decapitated hypertension is systolic blood pressure at a relatively low level, including patients who previously had hypertension. This condition is commonly found in severe HF because of decreased left ventricular ejection fraction and cardiac output.^2^, ^17^, ^23^, ^24^

CAD can damage myocardial cells because of ischemic processes, leading to HF in the future. At the same time, excessive RAAS activation and irregular ventricular contraction in AF also lead to diastolic dysfunction. All of these conditions can generate HF.^1^ We discovered that CAD did not significantly increase the likelihood of new‐onset HF [OR 1.66 (95% CI 0.70–3.93); *p* = .246], similar to a study by Eggimann et al.,^13^ despite three previous studies stating the opposite result.^2^, ^4^, ^25^

Another risk factor evaluated in our study was diabetes, which is a major risk factor for cardiovascular diseases, including AF, ischemic heart disease, and HF.^26^ Diabetes did not significantly increase the likelihood of new‐onset HF in AF patients in bivariate analysis, although the OR was >1 [OR 1.68 (95% CI 0.71–3.97); *p* = .228]. This finding was consistent with the 2017 ORBIT‐AF registry study,^27^ whereas other research (Krisai, et al. and Polovina, et al.) suggested the contrary.^2^, ^28^ However, we did not assess patients' control of their diabetes, which may have influenced the results such that diabetes was not statistically significant as a risk factor for new‐onset HF.

Obesity increases HF risk because of a pro‐inflammatory state and increased blood volume, which in turn leads to left ventricular hypertrophy. Moreover, increased BMI has also been associated with left atrium dilatation, with increased pressure and volume.^1^, ^29^, ^30^ On the contrary, based on bivariate analysis, we found that obesity was not a risk factor for new‐onset HF in AF patients [OR 1.73 (95% CI 0.78–3.84); *p* = .138]. This finding aligned with a meta‐analysis^2^ and another ORBIT‐AF registry study.^31^ The obesity paradox, a term that refers to the inverse relationship between BMI and adverse patient outcomes including HF, CAD, AF, and hypertension, probably caused the result to lack statistical significance.^32^ There are several hypothesized mechanisms in the obesity paradox, including lower increases of plasma renin and angiotensin as a response to stress giving a better outcome and providing greater metabolic reserves to counteract increased catabolic stress.^25^, ^31^

Smoking increases the risk of HF by reducing oxygen uptake capacity and causing coronary vasoconstriction, which leads to further myocardial ischemia. Moreover, smoking can also accelerate atherosclerosis.^30^ In our study, however, bivariate analysis showed that smoking had no significant relationship with new‐onset HF in AF patients [OR 2.22 (95% CI 0.99–4.96); *p* = .049], consistent with studies by Krisai et al., Eggimann et al., and Pandey et al.^2^, ^17^, ^33^

## 5 CONCLUSIONS

CKD and increased LAVI were associated with an increased risk of new‐onset HF in AF patients. However, hypertension, diabetes, CAD, smoking, and obesity were not associated with an increased risk of new‐onset HF in our study. Our findings suggest that clinicians should conduct routine screenings for comorbid CKD by laboratory and echocardiographic examinations to determine LAVI in AF patients. Controlling risk factors more tightly may help prevent new‐onset HF. In addition, early detection of new‐onset HF is important so that HF can be treated earlier.

## AUTHOR CONTRIBUTIONS

All authors formulated the research from concept to study design and methodology. Resultanti Irwan Muin organized project administration and resources. Resultanti Irwan Muin and Kuntjoro Harimurti analyzed and created visualizations of the results. Resultanti Irwan Muin, Muhammad Yamin, Ika Prasetya Wijaya, and Kuntjoro Harimurti wrote and edited the manuscript. Muhammad Yamin, Ika Prasetya Wijaya, Kuntjoro Harimurti, Hamzah Shatri, Cosphiadi Irawan, and Pradana Soewondo supervised the preparation of the manuscript. Muhammad Yamin, Ika Prasetya Wijaya, and Kuntjoro Harimurti revised the manuscript. All authors gave their final approval of the version to be published and were in agreement to be accountable for all aspects of the work.

## FUNDING INFORMATION

No specific funding was received from anybody in the public, commercial, or not‐for‐profit sectors to carry out the work described in this article.

## CONFLICT OF INTEREST STATEMENT

The authors declare that there are no conflicts of interest for this article.

## ETHICS APPROVAL STATEMENT

This research was approved by The Health Research Ethics Committee, Faculty of Medicine, Universitas Indonesia, with an approval number of KET‐1762/UN2.F1/ETIK/PPM.00.02/2023.

## Data Availability

Data is available upon request due to privacy and ethical restrictions. The findings of this study can be supported by data available from the corresponding author upon request.
